# Simulated Learning, Real Emotions: The Impact of Simulation-Based Education on Nursing Students’ Stress Levels During Objective Structured Clinical Examination: A Longitudinal Observational Cohort Study

**DOI:** 10.3390/nursrep15080307

**Published:** 2025-08-21

**Authors:** Kazimiera Hebel, Aleksandra Steliga, Katarzyna Lewandowska, Mikolaj Majkowicz, Aleksandra Goral-Kubasik, Monika Buzanowska, Mateusz Lammek, Andrew Sykes, Mateusz Puslecki, Przemyslaw Kowianski

**Affiliations:** 1 Institute of Health Sciences, Pomeranian University of Slupsk, 64 Bohaterow Westerplatte St., 76-200 Slupsk, Poland; aleksandra.steliga@upsl.edu.pl (A.S.); mikolaj.majkowicz@upsl.edu.pl (M.M.); aleksandra.goral-kubasik@upsl.edu.pl (A.G.-K.); monika.buzanowska@upsl.edu.pl (M.B.); przemyslaw.kowianski@upsl.edu.pl (P.K.); 2 Department of Anesthesiology and Intensive Care Nursing, Medical University of Gdansk, 7 Debinki Street, 80-211 Gdansk, Poland; kalewandowska@gumed.edu.pl; 3 Faculty of Social Sciences, Institute of Psychology, University of Gdansk, 4 Bazynskiego Street, 80-309 Gdansk, Poland; mateusz.lammek@ug.edu.pl; 4 Royal Brompton & Harefield Hospitals, Sydney Street, London SW3 6NP, UK; a.sykes@rbht.nhs.uk; 5 Department of Medical Rescue, Poznan University of Medical Sciences, 7 Rokietnicka Street, 60-806 Poznań, Poland; mateuszpuslecki@o2.pl

**Keywords:** simulation-based education, nursing students, stress, exam

## Abstract

**Background**: Simulation-based education (SBE) is a key component of nursing training. It enables students to apply theoretical knowledge in practice, expand their clinical understanding, develop critical thinking, improve communication skills, and build self-confidence. Increasing the number of simulation hours in nursing curricula may enhance students’ ability to manage stress in clinical settings. **Methods**: This was a cross-sectional comparative study involving 113 nursing students during a practical exam. Participants graduated in three consecutive years: 2020, 2021, and 2022. SBE was introduced into the curriculum in 2020, resulting in varying levels of simulation experience. Stress and anxiety markers were measured during the exam and compared across groups. **Results**: Greater simulation experience was associated with lower heart rate (*p* = 0.007), lower diastolic blood pressure (*p* < 0.001), and lower cortisol levels (*p* < 0.001). Students with two years of simulation training reported the lowest perceived stress (*p* = 0.031). However, anxiety levels remained high across all groups and did not differ significantly. **Conclusions**: The study showed that OSCEs are associated with elevated stress in nursing students. Students with greater exposure to simulation-based education had significantly lower stress and anxiety indicators. SBE appears to reduce stress and improve students’ preparedness for clinical assessments.

## 1. Introduction

The primary aim of nursing education is to equip graduates with the knowledge and skills required to provide safe and effective patient care [1]. Practical components of vocational training are delivered in clinical learning environments, which include hospitals, outpatient clinics, and medical simulation centers [2,3]. Simulation-based classes offer a standardized and reproducible setting in which students can develop clinical, technical, cognitive, and behavioral competencies using both task trainers and standardized patients [1]. Numerous studies have demonstrated that simulation-based education (SBE) is a fundamental component of nursing training. It facilitates the transfer of theoretical knowledge into clinical practice, enhances critical thinking, strengthens communication skills, and fosters self-confidence and learning satisfaction among students [4,5].

### 1.1. OSCE Exam

The medical simulation environment enables the objective assessment of nursing students’ clinical competence through the use of the Objective Structured Clinical Examination (OSCE) [6]. The OSCE is a widely recommended method for evaluating clinical skills in nursing education and represents an integral and widely adopted component of summative assessment across medical faculties [7,8,9]. In this examination format, students rotate through a series of simulation stations, performing tasks at varying levels of fidelity. Each station is designed to assess a specific competency using standardized checklists [10]. While organizing and administering an OSCE is resource-intensive, its educational value is considered to outweigh both financial and logistical costs [11]. Compared to traditional assessment formats, OSCEs may induce higher levels of stress and anxiety in students [6,12]. Although numerous studies highlight that simulation-based teaching and examination formats enhance graduates’ readiness for independent clinical practice, they also show that such formats can provoke significant psychological stress [6,13,14,15,16,17,18,19].

Various interventions aimed at reducing anxiety during OSCEs have also been undertaken, some of which have proven effective in certain studies and translated into improved exam performance [20]. However, the latest findings from 2025 indicate that a one-time intervention involving relaxation, mindful breathing, and positive reinforcement significantly lowers anxiety levels but does not have a substantial impact on exam outcomes [21]. Contrasting conclusions were presented by researchers analyzing a group of pharmacy students, who suggest that supportive measures should focus on factors beyond merely reducing anxiety [22]. The topic of stress and anxiety associated with examinations remains an area of intensive research, with ongoing efforts to both alleviate stress and increase pass rates. This presents a considerable challenge for educators, as demonstrated by a 2024 study highlighting the importance of exam organization quality in reducing stress and improving overall average scores [23].

### 1.2. Psychological Stress Among Students in Academic Settings

In physiological terms, stress is the body’s reaction to an external stimulus interpreted as a potential threat or stressor. Stress induces activation of the sympathetic nervous system and the hypothalamic–pituitary–adrenal axis. This, in turn, results in increased production of cortisol, adrenaline, and noradrenaline, leading to an increase in blood pressure and heart rate [24]. The major stressors for students include the process of preparing for the exam, taking the exam, and anxiety over being evaluated [25]. The notion of stress is usually associated with negative feelings. Nevertheless, small amounts of stress may be beneficial for students, motivating them to achieve better results. However, it is difficult to draw a line between the optimal level of stress, called eustress, and the excessively high level of stress, called distress [26]. It is also important to identify subjective and external factors affecting the severity of stress during simulation classes or examinations.

The experience of education through simulations alters students’ emotional activation [13]. Emotions related to academic achievement have a key impact on students’ academic performance and their successes and failures [14]. One of the emotions associated with skill acquisition and verification of students’ learning outcomes in medical simulation is anxiety [17]. Anxiety is an autonomic response manifesting as a sense of uneasiness and tension as a result of anticipating a real or imagined threat. It is a subjective feeling causing discomfort in students [18,19]. Studies have shown that this is mainly associated with a lack of experience in simulation-based education [17]. The presence of anxiety during classes can potentially lead to a reduced ability to memorize information, resulting in more mistakes made by students and less success achieved in the educational process [17]. The use of curricula which include simulation-based education has consistently reduced anxiety levels in novice nursing students and improved their self-confidence [15].

### 1.3. Simulation in Nursing Education in Poland

In Poland, education in medical faculties is regulated by education standards approved by the Minister of Higher Education. These standards must also be in line with the guidelines of the Minister of Health and EU Directives. In light of these regulations, there is an ongoing discussion among experts about specifying a minimum number of hours of practical simulation training to be included in the education standard. The primary goal of such a provision is to ensure an opportunity for acquiring technical, clinical thinking, and decision-making skills under conditions of psychological comfort for the student, ultimately contributing to improved patient safety.

The aim of this study was to assess physiological and psychological stress markers among nursing students during the OSCE practical exam and to compare these indicators based on students’ prior exposure to simulation-based education. This research responds to the growing need for evidence on how simulation training influences emotional responses in clinical assessment contexts, particularly in countries where SBE has only recently been introduced into nursing curricula.

## 2. Materials and Methods

### 2.1. Study Design

This was a cross-sectional comparative study involving three cohorts of students to assess perceived stress and the use of simulation-based learning among undergraduate nursing students in Poland. The study was conducted according to the Strengthening the Reporting of Observational Studies in Epidemiology (STROBE) criteria [27].

### 2.2. Participants

The study participants were nursing students (*n* = 113) with varying experience in SBE. The students were divided into three groups: group 1—students with no experience in SBE (graduating class of 2020, *n* = 42); group 2—students with one-year experience in SBE (graduating class of 2021, *n* = 33); group 3—students with two-year experience in SBE (graduating class of 2022, *n* = 38). Participation in the study was voluntary. Participants received no incentives or payment for their involvement. Each student received detailed written information about the study and subsequently provided written informed consent to participate.

### 2.3. Measurement Instruments

The State-Trait Anxiety Inventory (STAI) was developed by Spielberger, Gorsuch, and Lushene [28]. The authors of the Polish version of the tool are [29]. STAI measures anxiety in adolescents and adults. The questionnaire is composed of two parts: 1. Anxiety understood as a state in a given moment (STAI X-1); 2. Anxiety perceived as a trait (STAI X-2). In the study, we used the state anxiety scale measuring anxiety in a given moment (STAI X-1). The raw scores in this scale range from 20 to 80, with higher scores denoting greater anxiety. The raw scores can be converted to standardized sten scores (0–10) or percentile scores (0–100). For the purpose of this study, the raw scores were also presented as stens. The internal consistency coefficients (Cronbach’s alpha) for adults on both scales are satisfactory and fall within the range of 0.89–0.92 for state anxiety and 0.76–0.90 for trait anxiety [29].

The Perceived Stress Scale (PSS-10) developed by Cohen and adapted for the Polish setting by Jurczyński [30] is used to assess perceived stress levels in a given life situation over the previous month. The PSS score obtained by subjects pertains to psychological and somatic symptoms. The total PSS score ranges between 0 and 40, with higher scores denoting higher stress levels. It is possible to convert the raw scores into stens (0–10). The reliability of the Polish version of the questionnaire was found to be satisfactory. The internal consistency measured with Cronbach’s alfa is 0.86, which is similar to the value observed for the original version, i.e., 0.84–0.86 among three groups of subjects [30].

### 2.4. Research Procedure

The study was conducted between 15 June 2020 and 15 July 2022 at a university medical simulation center in Poland. The research was carried out over a period of three consecutive years. All final-year students were invited to participate, and each provided informed consent. Examination conditions were standardized across all groups. In each of the three years, the examination was conducted under identical protocols and within the same simulation environment. Prior to the main assessment, students from all three cohorts took part in a preliminary pre-OSCE examination.

The study was conducted on the day of the diploma exam, which concluded a three-year Bachelor of Science in Nursing program and constituted the final stage of verifying the level of learning outcomes and professional competence achieved by the students. Based on their experience in SBE, the students were divided into three groups. In the 1st group were students with no experience in simulation; the study program did not provide SBE hours. In the 2nd group were students with one-year experience in SBE. In this group, the study program included 108 SBE hours for the 3rd year of study. In the 3rd group were students with two-year experience in SBE. In this group, the study program included 206 SBE hours for the 2nd and 3rd year of study. All students passed the practical exam in OSCE format in the same simulation environment. During the OSCE, all students had to perform three instrumental procedures (low-fidelity) and manage three clinical cases scenarios (high-fidelity) for the assessment of non-technical skills such as critical thinking, decision-making, and interpersonal communication. In the 1st group of students who had not previously studied using high fidelity simulators, standardized patients were used during the OSCE exam. All students knew the simulation environment and participated in the pre-OSCE before the final exam.

The study protocol involved first measuring vital signs and collecting a blood sample, followed by the completion of questionnaires assessing anxiety and stress. Subsequently, the students proceeded to take the examination. The entire data collection procedure lasted 30 min, as specified in the research protocol. Therefore, data collection for all students was initiated 30 min prior to the start of the OSCE examination. The physiological stress markers included the main vital signs such as systolic blood pressure (BP_SYS) and diastolic blood pressure (BP_DIA) measured in mmHg, and heart rate (HR) measured in bpm. The biochemical marker was the serum cortisol level (CORT) measured in µg/dL. The psychological markers, i.e., anxiety and stress, were assessed using standardized tools, namely the State-Trait Anxiety Inventory (STAIX-1) and the Perceived Stress Scale (PSS-10), respectively. The psychological test results were analyzed by a researcher with a degree in psychology.

A qualified nurse took blood samples and measured the vital signs. The students did not suffer from any chronic diseases and did not report any health problems. They had an overnight rest, were in a fasting state, and did not engage in exercise before the study. The blood samples were transferred to a laboratory. Cortisol levels were measured using a Roche Cobas e 411 machine (Hitachi High-Tech Corporation, 1-17-1 Toranomon, Minato-ku, Tokyo, Japan). The Elecsys® Cortisol II assay used to quantify cortisol levels in serum is the second-generation assay (Roche Diagnostics GmbH, Mannheim, Germany). All assays were solid-phase competitive binding immunoassays using chemiluminescent detection. The cobas e 411 analyzer is a fully automated analyzer that uses a patented ElectroChemiLuminescence (ECL) technology for immunoassay analysis.

The vital signs were measured using a validated electric blood pressure monitor in a sitting position. The students completed paper-based questionnaires in specially prepared silent rooms.

### 2.5. Ethical Consideration

The study was conducted in accordance with the protocol approved by the Bioethics Committee at the District Medical Chamber in Gdansk (No. KB-12/20) and adhered to applicable legal regulations as well as the bioethical principles outlined in the Declaration of Helsinki.

### 2.6. Data Analysis

Descriptive statistics including mean, median, standard deviation, frequency, percentage, and maximum and minimum (range) were used in the study. The Shapiro–Wilk W test was used to assess the empirical distribution of data. Data that did not follow a normal distribution were analyzed with non-parametric tests such as Spearman’s rho correlation matrix, the Kruskal–Wallis test and one-way analysis of variance (ANOVA). The threshold of statistical significance for all tests was set at *p* = 0.05. The calculations were made with the use of StatSoft STATISTICA version 13.3 (Poland, Cracow, 2020).

## 3. Results

### 3.1. Study Group Characteristics

The study group included 103 females (91.15% of the total number of participants) and 10 males (8.85%). The mean age of the participants was 25 years, with an age range of 21–50 years. The mean age of students in group 1 was 27.05 years, in group 2 was 24.24 years, and in group 3 was 24.74 years. The one-way analysis of variance showed that there were no statistically significant differences between the groups in terms of age: F(2.110) = 2.09; *p* = 0.128. The demographic characteristics of the participants indicated overall homogeneity within the study population.

### 3.2. Physiological Markers of Stress

Systolic blood pressure (BP_SYS) in the entire study group ranged from 107 to 162 mmHg, with a mean value of M = 131.04 mmHg. The mean BP_SYS values in groups 1–3 were as follows: M = 134.31, M = 130.21, and M = 128.16, respectively. The BP_SYS values tended to decrease as the experience in SBE increased, but the differences were not statistically significant (*p* = 0.073) (Table 1).

Diastolic blood pressure (BP_DIA) ranged from 56 to 127 mmHg, with a mean value of M = 79.05 mmHg. The mean BP_DIA value in group 1 was M = 85.30. In groups 2 and 3, it was M = 78.45 and M = 72.58 mmHg, respectively. We found that there were statistically significant differences (*p* < 0.001) in the BP_DIA results between the groups, with the values decreasing as the experience in SBE increased (Table 1).

Heart rate (HR) in the entire study group ranged from 57 to 140 bpm, with a mean value of M = 94.81bpm. There were statistically significant differences (*p* = 0.007) in the HR results between the groups (Table 1). The post-hoc tests (with the Bonferroni correction) for ANOVA showed that in terms of HR, group 1 differed statistically significantly from group 2 (*p* = 0.012) and group 3 (*p* = 0.004). There were no statistically significant differences between group 2 and group 3. The summary of the results of the post-hoc analysis is presented in the last table.

### 3.3. Biochemical Markers of Stress

The blood serum test results showed that the level of cortisol (CORT) ranged from 4.54 to 52.77 ug/dL (M = 17.34). We compared these values with the reference range for healthy populations specified by the manufacturer, Roche: morning hours 6 10 a.m.: 6.02 18.4 µg/dL. The mean cortisol level in group 1 exceeded the normal range (M = 21.93). We demonstrated a statistically significant difference in the CORT value between groups (*p* < 0.001) (Table 2). The post-hoc tests (with the Bonferroni correction) showed that the level of cortisol in group 1 was clearly higher than in group 2 (*p* < 0.001) and group 3 (*p* < 0.001). In groups 2 and 3, cortisol level was similar (*p* = 0.701). 

### 3.4. Psychological Markers of Stress

In the study, we used the state anxiety scale measuring anxiety in a given moment (STAI X-1). The raw scores ranged from 22 to 72, with a mean score of M = 48.96 (Table 3). The anxiety scores in the groups tended to decrease as the experience in SBE increased, but the differences were not statistically significant (*p* = 0.154).

The overall stress score measured with PSS-10 was high and ranged from 1–29, with a mean value of M = 19.05. The level of stress in the groups varied significantly (*p* = 0.031) (Table 3). The post-hoc tests (with the Bonferroni correction) showed that the level of perceived stress in group 3 was clearly lower than in group 2 (*p* = 0.016) and group 1 (*p* = 0.031). In groups 1 and 2, stress level was similar (*p* = 0.694) (Table 4).

We identified the extreme stress scores (1st and 4th percentiles) in the entire study group. The lowest PSS scores (1st percentile) were obtained by 30 students (26.5% of the total number of participants), including 14 students with a two-year experience in SBE. That group also included 11 students with no experience and 5 students with one-year experience in SBE. In turn, the highest PSS scores (4th percentile) were obtained by 31 students (27.4%), including 13 students with no experience in SBE. That group also included 11 students with one-year experience and 7 students with two-year experience in SBE.

## 4. Discussion

This study provides evidence that increased exposure to simulation-based education (SBE) is associated with reduced stress and anxiety during high-stakes practical assessments, such as the Objective Structured Clinical Examination (OSCE). These findings are particularly relevant in the context of ongoing discussions in Poland regarding the optimal number of simulation hours to be included in nursing curricula. Expanding SBE may enhance students’ clinical readiness while helping them manage performance-related stress.

Medical simulation exposes students to a realistic simulation scenario, allowing them to experience difficult situations in a learning environment [5]. An analysis of the available literature showed that according to students, stress levels during an OSCE are influenced by such factors as time pressure, little or no knowledge of the simulation environment, stressful and tense atmosphere, and the awareness of being evaluated [11,31,32]. Our results are consistent with these observations but further extend them by showing that students with more extensive SBE experience exhibit significantly lower physiological stress responses.

Evidence from studies in this domain demonstrates that the integration of simulation-based methods into nursing curricula contributes to the enhancement of educational standards [33]. Simulation has also been shown to be an effective strategy for reducing anxiety and increasing self-confidence in nursing students as compared to conventional teaching strategies [34]. Our study also demonstrated a positive impact of SBE on stress reduction in students during a practical exam. We observed that although anxiety and stress levels experienced at the time of the practical exam were above average in all the groups, they tended to decrease along with greater experience in SBE. Stress levels were lowest in students with the largest number of hours of practical simulation-based education in their training curriculum. Other authors observed similar results, demonstrating that any form of examination induces anxiety in the subjects [35,36]. Researchers have pointed to factors that reduce anxiety or stress during a practical exam, such as offering students an opportunity to participate in a pre-OSCE [34]. Furthermore, studies by Erfanian and Khadivzadeh (2011) have shown that students present lower levels of anxiety during an OSCE as compared to real-life situations associated with the fear of harming a real patient. As they experience simulations during the subsequent stages of training, students not only gain experience and become accustomed to the simulation environment but also learn and understand the purpose of simulations and assessment methods [34].

Sánchez-Conde et al. (2021) conducted a study analyzing the autonomic response to a stressful situation, i.e., an OSCE [37]. They found that the heart rate response in nursing students was consistent with the anticipatory anxiety response and was highest at the beginning of the exam and lowest during the break and after the exam. Our study confirmed that physiological parameters such as HR and BP were elevated in all the students, indicating the presence of stress. However, we found that the group of students with the longest experience in SBE displayed significantly lower HR values than students with no such experience. Sanchez-Conde et al. (2021), in their study, conducted three HR measurements—before, during, and after the OSCE exam [37]. In contrast, in our study, we performed a single measurement of the vital signs—immediately before the start of the exam, which is considered the moment of the highest emotional tension. Repeated measurements of these signs during the exam would have allowed for assessing changes in the autonomic response in the consecutive OSCE stages.

Cortisol is considered an important stress marker [38]. Our findings complement previous research by Powell et al. (2012) on the cortisol awakening response (CAR), which has been linked to coping with daily stress [38]. In our study, we observed considerable variability in cortisol levels among participants. Notably, the mean cortisol levels in the group without experience in simulation-based education (SBE) exceeded the normal range, suggesting a heightened physiological stress response. In contrast, participants with one- and two-year experience in SBE showed similar cortisol levels, which may reflect a more adaptive stress regulation. These results align with the notion that experience and familiarity with demanding situations, such as simulations, may contribute to more effective stress coping mechanisms, as indicated by stable cortisol responses.

Adibone Emebigwine et al. (2023) showed that, in order to reduce stress levels and improve learning outcomes, it is reasonable to allow students to participate in a mock exam (so-called pre-OSCE), define the timeframe for each station, provide training in time management, and foster positive attitudes among examiners [32]. Similarly, the results of our study demonstrate that the main advantage of increasing the number of hours dedicated to simulation-based education (SBE) is the opportunity it provides for students to acquire technical skills, clinical reasoning, and decision-making abilities in an environment that ensures psychological comfort for the learner and safety for the patient. The ability to work under stress and time pressure, to respond to abrupt situational changes and make decisions are key skills for medical professionals.

In terms of practical application, our findings highlight the rationale for increasing the number of hours allocated to medical simulation in nursing education programs. We confirmed that having experienced stressful situations in a medical simulation setting reduces anxiety and stress in students. Consequently, students can develop skills and competences that will allow them to manage their emotions. This may also help to improve their self-confidence in real-life settings, thus increasing their comfort, and work and patient safety. Our findings can be used by decision-makers and planners—authors of education curricula for health professions—to effectively and swiftly introduce more hours of medical simulation as an anxiety and fear management program.

## 5. Conclusions

The study confirmed that participation in an OSCE induces elevated levels of stress and anxiety in nursing students, as evidenced by increased physiological and biochemical markers. However, the intensity of the stress response varied significantly depending on students’ prior exposure to simulation-based education (SBE). Students with greater SBE experience demonstrated significantly lower perceived stress (PSS-10), heart rate, and cortisol levels compared to those without SBE training. These findings support the value of simulation-based education in enhancing students’ stress resilience and preparedness for high-stakes clinical assessments.

## 6. Implications for Practice

The aim of our study was to assess stress and anxiety levels in nursing students prior to a practical clinical examination, thereby identifying emotional factors that may influence students’ ability to manage stressful scenarios.

Clinical practice: Our findings confirm that incorporating SBE into nursing curricula can help reduce stress and anxiety during high-pressure evaluation settings. Enhanced simulation training may better prepare students for real-life clinical interventions by creating psychologically safe, yet challenging environments for skill development.

Management: These results provide evidence to support educational policymakers and curriculum developers in implementing or expanding SBE hours as part of national training standards. Simulation-based training may serve as a structured strategy for managing anxiety and fear in clinical education.

Education: All forms of examination tend to evoke stress and anxiety in students. However, the positive impact of SBE suggests that introducing simulation earlier in the educational pathway—well before the OSCE—can help students gain valuable experience, thereby improving their readiness to cope with emotionally demanding assessments.

## 7. Limitations of the Study

This study has several limitations. Stress markers were measured 30 min before the OSCE, a point likely associated with anticipatory anxiety. Future research should include repeated measurements—before, during, and after the exam—to capture the dynamic nature of stress responses. Additionally, incorporating a control group of students undergoing a non-terminal OSCE (e.g., at the end of the first year) could help distinguish between exam-related and graduation-specific stress. Future studies should also explore students’ subjective experiences by identifying their perceived sources of stress and anxiety. Finally, participant feedback on how well simulation training translated into real clinical practice would offer a valuable complementary perspective.

## Figures and Tables

**Table 1:** Physiological markers of stress according to experience in simulation-based education.

Variable		Total (*n* = 113)	Group Without Simulation Experience (*n* = 42)	Group with One-Year Simulation Experience (*n* = 33)	Group with Two-Year Simulation Experience (*n* = 38)	*p*-Value
BP_SYS	Range	107–162	113–156	109–162	107–158	0.073 ^1^
	Me	128	134.5	128	127	
	M	131.04	134.31	130.21	128.16	
	SD	12.31	12.31	12.65	11.43	
BP_DIA	Range	56–127	60–127	61–94	56–92	<0.001 ^1^
	Me	79	83	78	72	
	M	79.05	85.38	78.45	72.58	
	SD	11.01	11.25	8.51	8.63	
HR	Range	57–140	57–137	69–113	63–140	0.007 ^2^
	Me	93	103	88	88	
	M	94.81	101.00	91.69	90.68	
	SD	16.39	18.65	12.39	15.04	

^1^ Kruskal–Wallis test, ^2^ test F for one-way analysis of variance; BP_SYS—systolic blood pressure; BP_DIA—diastolic blood pressure; HR—heart rate; Me—median; M—mean; SD—standard deviation.

**Table 2:** Biochemical markers of stress according to experience in simulation-based education.

Variable		Total (*n* = 113)	Group Without Simulation Experience (*n* = 42)	Group with One-Year Simulation Experience (*n* = 33)	Group with Two-Year Simulation Experience (*n* = 38)	*p*-Value
CORT	Range	4.54–52.77	7.63–52.77	4.54–23.95	6.53–36.17	<0.001 *
	Me	15.1	19.67	14.01	13.99	
	M	17.34	21.93	14.21	14.99	
	SD	9.08	11.5	4.75	6.72	

* Kruskal–Wallis test; CORT—level of cortisol in blood serum; Me—median; M—mean; SD—standard deviation.

**Table 3:** Psychological markers of stress according to experience in simulation-based education.

Variable			Total (*n* = 113)	Group Without Simulation Experience (*n* = 42)	Group with One-Year Simulation Experience (*n* = 33)	Group with Two-Year Simulation Experience (*n* = 38)	*p*-Value
STAI X-1	Raw score	Range	22–72	22–69	29–67	27–72	0.154 ^1^
		Me	50.00	53.50	48	48.50	
		M	48.96	50.17	47.73	48.68	
		SD	10.11	10.93	8.85	10.29	
	Sten	Range	2–10	2–10	3–10	3–10	
		Me	8.00	9.0	8.00	8.00	
		M	7.83	7.95	7.67	7.84	
		SD	1.98	2.17	1.76	1.98	
PSS 10	Raw score	Range	1–29	8–29	10–29	1–27	0.031 ^2^
		Me	20.00	21.00	21.00	18.00	
		M	19.05	19.86	20.39	17.00	
		SD	5.99	5.88	5.47	6.15	
	Sten	Range	2–9	3–9	4–9	2–9	
		Me	7.00	7.00	7.00	6.00	
		M	6.53	6.59	6.7	5.79	
		SD	1.79	1.81	1.67	1.77	

^1^ Kruskal–Wallis test, ^2^ test F for one-way analysis of variance; Me—median; M—mean; SD—standard deviation.

**Table 4:** Post hoc analysis: statistically significant differences between groups.

	HR		PSS-10		CORT	
	Mean Difference	*p*-Value	Mean Difference	*p*-Value	Mean Difference	*p*-Value
Group 1 vs. Group 2	9.31	0.012	−0.53	0.694	7.72	0.001
Group 1 vs. Group 3	10.32	0.004	2.86	0.031	6.94	0.001
Group 2 vs. Group 3	1.01	0.674	3.39	0.016	−0.78	0.701

Group 1 with no simulation experience; Group 2 with one year of simulation experience; Group 3 with two years of simulation experience.

## Data Availability

Data are available from the first author upon reasonable request.

## Public Involvement Statement:

No public involvement in any aspect of this research.

## Guidelines and Standards Statement:

This manuscript was drafted against the STROBE (Strengthening the Reporting of Observational Studies in Epidemiology) guidelines for observational research.
